# STAT4 controls GM-CSF production by both Th1 and Th17 cells during EAE

**DOI:** 10.1186/s12974-015-0351-3

**Published:** 2015-06-30

**Authors:** Ian L. McWilliams, Rajani Rajbhandari, Susan Nozell, Etty Benveniste, Laurie E. Harrington

**Affiliations:** Department of Cell, Developmental and Integrative Biology, University of Alabama at Birmingham, 845 19th Street South, BBRB 471, Birmingham, AL 35294 USA

**Keywords:** STAT4, EAE, MS, Th17, Th1, GM-CSF

## Abstract

**Background:**

In experimental autoimmune encephalomyelitis (EAE), a mouse model of multiple sclerosis, mice genetically deficient in the transcription factor signal transducer and activator of transcription 4 (STAT4) are resistant to disease. In contrast, deletion or inhibition of the Th1-associated cytokines IL-12 or IFNγ which act upstream and downstream of STAT4, respectively, does not ameliorate disease. These discordant findings imply that STAT4 may act in a non-canonical role during EAE. Recently, STAT4 has been shown to regulate GM-CSF production by CD4 T cells and this cytokine is necessary for the induction of EAE. However, it is not known if STAT4 controls GM-CSF production by both Th1 and Th17 effector CD4 T cells.

**Methods:**

This study utilized the MOG_35–55_ peptide immunization model of EAE. Intracellular cytokine staining and novel mixed bone marrow chimeric mice were used to study the CD4 T cell-intrinsic role of STAT4 during disease. STAT4 chromatin-immunoprecipitation (ChIP-PCR) experiments were performed to show STAT4 directly interacts with the *Csf2* gene loci.

**Results:**

Herein, we demonstrate that STAT4 controls CD4 T cell-intrinsic GM-CSF production by both Th1 and Th17 CD4 T cells during EAE as well as in vitro. Importantly, we show that STAT4 interacts with the *Csf2* locus in MOG_35–55_-activated effector CD4 T cells demonstrating direct modulation of GM-CSF.

**Conclusions:**

Overall, these studies illustrate a previously unrecognized role of STAT4 to regulate GM-CSF production by not only Th1 cells, but also Th17 effector CD4 T cell subsets during EAE pathogenesis. Critically, these data highlight for the first time that STAT4 is able to modulate the effector profile of Th17 CD4 T cell subsets, which redefines our current understanding of STAT4 as a Th1-centric factor.

**Electronic supplementary material:**

The online version of this article (doi:10.1186/s12974-015-0351-3) contains supplementary material, which is available to authorized users.

## Background

Multiple sclerosis (MS) is a demyelinating autoimmune disease characterized by the presence of CD4 T cells in inflammatory lesions within the central nervous system (CNS) [1, 2]. Studies using the mouse model of MS, experimental autoimmune encephalomyelitis (EAE), have demonstrated that the Th1 and Th17 CD4 T cell subsets are associated with disease onset and that both subsets are capable of causing disease. Interestingly, while both Th1 and Th17 cells can initiate disease, the mechanisms by which these cells mediate inflammation and characteristics of the disease are different [3–5]. For instance, Th1 cells preferentially migrate to the spinal cord and recruit macrophages to sites of inflammation, whereas Th17 cells primarily infiltrate the brain and recruit neutrophils. Nevertheless, it is possible that Th1 and Th17 CD4 T cells share properties that contribute to pathogenicity, and defining these potential commonalities may lead to new therapeutic targets.

A recent genome-wide association study (GWAS) identified a polymorphism in the signal transducer and activator of transcription 4 (STAT4) gene that is associated with MS susceptibility [6]. STAT4 is a member of the STAT family of transcription factors and is a Th1 transcriptional regulator [7, 8]. STAT4, when activated by IL-12, results in the development of Th1 cells and the production of the hallmark Th1 cytokine IFNγ. Paradoxically, neither IL-12 nor IFNγ is required for EAE, while STAT4 is essential. This highlights an important, unknown function for STAT4 during chronic CNS inflammation that is independent of the classic Th1 pathway [9–15]. During Th1 differentiation, STAT4 is necessary to establish the genomic landscape, which then allows other transcription factors to bind Th1 lineage-associated genes [7, 16, 17]. It remains unclear if STAT4 instructs the epigenetic landscape in effector CD4 T cells outside of the Th1 lineage. While Th17 differentiation is not contingent on STAT4, the role of this molecule in Th17 plasticity, and potentially Th17 gene expression, remains controversial [18–20]. Hence, STAT4 may function in Th17 cells during EAE, possibly by shaping the accessibility and expression of encephalogenic genes.

In addition to IFNγ, the Th17 prototypic cytokine IL-17A is also dispensable for EAE, raising the question as to how these CD4 T cells mediate disease and if these effector subsets share an encephalogenic molecule [15, 21]. Both Th1 and Th17 CD4 T cells produce GM-CSF, which has been demonstrated to be critical for EAE pathogenesis by both cell types [22, 23]. GM-CSF functions to activate microglia within the CNS as well as recruit and stimulate peripheral macrophages and dendritic cells during EAE [24, 25]. Recent studies show that STAT4 knockout (STAT4^−/−^) T cells had diminished GM-CSF production [26, 27]. These data suggest that STAT4 may regulate GM-CSF, which in turn drives the development of EAE. However, whether STAT4 acts in a CD4 T cell-intrinsic manner and if STAT4 regulates both Th1 and Th17-derived GM-CSF production during EAE were not determined.

In this study, we investigated the relationship between STAT4 and GM-CSF production during EAE. We demonstrate that STAT4 regulates GM-CSF expression by not only MOG-specific Th1 cells, but also Th17 and a population of GM-CSF+IFNγ−IL-17A− single-producing subset of cells during EAE. Coincident with a lineage-indiscriminate role for STAT4, we find that in vitro STAT4 functions to promote optimal GM-CSF production in CD4 T cells activated under non-polarizing, Th0 conditions. Using mixed bone marrow chimeric mice, we show that CD4 T cell-intrinsic STAT4 expression is important for GM-CSF production during EAE. Furthermore, STAT4 is able to directly bind to and regulate the *Csf2* promoter in encephalogenic CD4 T cells. Overall, this study illustrates that STAT4 directly regulates the transcription of GM-CSF and highlights a previously unrecognized role for STAT4 in the function of Th17 cells.

## Materials and methods

### Mice

C57BL/6J, B6.SJL-*Ptprca Pep3b*/BoyJ (WT CD45.1), and B6.129S7-*Rag1tm1Mom*/J (Rag1^−/−^) were purchased from the Jackson Laboratory. B6.STAT4^−/−^ (STAT4^−/−^) mice were generously provided by Dr. Mark Kaplan [28]. B6.*Ifng/Thy1.1* knock-in mice were described previously [29]. Both C57BL/6J and B6.*Ifng/Thy1.1* knock-in mice were used as wild-type (WT) controls. All animals were bred and maintained under specific pathogen-free conditions at the University of Alabama at Birmingham according to Institutional Animal Care and Use Committee regulations.

### Mixed bone marrow chimeric mice

Mixed bone marrow chimeric mice were generated as previously described [30]. Rag1^−/−^ mice were irradiated with a split dose of 1000 rad and reconstituted with CD5-depleted bone marrow by intravenous injection. The transferred bone marrow cells were a mixture of 50 % CD45.1 WT bone marrow and 50 % CD45.2 WT bone marrow (WT:WT) or 50 % CD45.1 WT bone marrow and 50 % CD45.2 STAT4^−/−^ bone marrow (WT:STAT4^−/−^). Recipient mice were maintained on antibiotic water for 6 weeks. Mice were immunized for EAE 10 weeks following reconstitution.

### EAE induction and clinical scoring

Age and sex matched mice between 8 and 12 weeks of age were induced for EAE by subcutaneous immunization with 50 μg MOG_35−55_ peptide (Biosynthesis) emulsified in CFA (150 μg *Mycobacterium tuberculosis*; Difco) and intraperitoneal (i.p.) administration of pertussis toxin (200 ng; List Biological Laboratories) on days 0 and 2. Disease was monitored daily by the following criteria: 0, no disease; 1, tail paralysis; 2.0, hind limb paresis; 3.0, complete hind limb paralysis; 4.0, forelimbs paralysis; and 5, moribund.

### Ex vivo stimulation

Single-cell suspensions of spinal cord, spleen, and inguinal LNs were prepared as previously described [31]. The following antibodies were used: anti-CD4 PerCP-Cy5.5/APC/eFluor 450/PE-Cy7 (eBioscience, clone RM4-5), anti-CD45.1 FITC (eBioscience, clone A20), anti-CD45.2 PerCP-Cy5.5/APC (eBioscience, clone 104), and anti-CD44 FITC (eBioscience, clone IM7). For intracellular staining, cells were reactivated with culture media (negative control) or 5 μM MOG_35−55_ peptide for 7 h with GolgiPlug (BD Biosciences) added for the final 4 h. The following intracellular antibodies were used in accordance with the manufacturer’s protocols: anti-IFNγ eFluor 450 (eBioscience, clone XMG1.2), anti-IL17A Alexa Fluor 647 (eBioscience, clone eBio17B7), anti-GM-CSF PE (BD Biosciences, clone MP1-22E9). A viability dye (Aqua, Life Technologies) was applied to exclude dead cells. Samples were acquired by using an LSRII flow cytometer (BD Biosciences) followed by data analysis using FlowJo version 9.x (Tree Star).

### Naïve CD4 T cell polarization and activation

Naïve CD4+CD25−CD45RB^hi^ (anti-CD25 PerCP-Cy5.5 (eBioscience, clone PC61.5); anti-CD45RB FITC (eBioscience, clone C363.16A)) T cells were sorted from WT and STAT4^−/−^ mice using a FACSAria cell sorter (BD Biosciences) and cultured in the presence of irradiated WT feeders in R10 containing 2.5 μg/ml anti-CD3 (clone 145-11) for 5–6 days. Conditions also contained the following: Non-polarizing (Th0) with anti-IL-12p40—10 μg/ml anti-IL-12p40 (clone C17.8); Th1—10 ng/ml rmIL-12 and 10 μg/ml anti-IL-4 (clone 11B11); Th17 (TGFβ1)—10 ng/ml rmIL-23, 20 ng/ml rmIL-6, 5 ng/ml rhTGFβ1, 10 μg/ml anti-IL-4, and 10 μg/ml anti-IFNγ (clone XMG1.2); Th17 (IL-1β)—10 ng/ml rmIL-23, 20 ng/ml rmIL-6, 5 ng/ml rmIL-1β, 10 μg/ml anti-IL-4, and 10 μg/ml anti-IFNγ. For intracellular staining, cells were stimulated with R10 only (negative control) or platebound anti-CD3 (10 μg/ml)/soluble anti-CD28 (1 μg/ml) for 7 h with GolgiPlug (BD Biosciences) added for the final 4 h. Neutralizing antibodies were obtained from UAB hybridoma facility.

### GM-CSF ELISA

Single-cell suspensions from draining inguinal lymph nodes were prepared and stimulated with either R10 only or 5 μg MOG_35−55_ peptide for 16 h. Supernatants were then collected and assessed for GM-CSF production by ELISA (eBioscience).

### RNA purification, cDNA synthesis, and real-time PCR

Positively selected CD4 T cells were isolated following stimulation. RNA collection, cDNA synthesis, and real-time PCR analysis were performed as described previously [31]. Primers used for indicated genes are as follows: *Csf2* forward: 5′-TGGAAGCATGTAGAGGCCATCA-3′; and *Csf2* reverse: 5′-GCGCCCTTGAGTTTGGTGAAAT-3′.

### Chromatin-immunoprecipitation PCR

ChIP assays were adapted from previously described methods [32]. Single-cell suspensions from pooled spleen and dLN were prepared and reactivated with either R10 or 5 μM MOG_35−55_ peptide for 5 h. CD4 T cells were purified, fixed, lysed with T cell lysis buffer (20 mM HEPES, pH 7.4), 150 mM NaCl, 1.5 mM MgCl_2_, 2 mM EGTA, 1 % Triton X-100, 12.5 mM β-glycerophosphate, 10 mM NaF, 1 mM Na_3_VO_4_), and then sonicated. Equal amounts of lysate were pre-cleared with BSA and SS-DNA-blocked protein A beads. Afterwards, 1/10th volume was removed and saved as “Input.” The remainder was immunoprecipitated with 4 μg of either STAT4 (Cell Signaling, clone C46B10) or Ser-2-Pol II CTD (Covance, clone H5) antibodies, and the immune complexes were absorbed with BSA and SS-DNA-blocked protein A beads (Upstate Cell Signaling Solutions, Charlottesville, VA). Immunoprecipitated DNA was analyzed by qRT-PCR using Sybr Green reagents. Primers used for indicated promoter regions are as follows: *Csf2* forward: 5′-GGTCTCCTCAGTGGGAGTCTGT-3′; *Csf2* reverse: 5′-GGGGTTTGGGAGATACTGAGTG-3′; *Ifng* forward: 5′-TTTCTGGGCACGTTGACCCT-3′; and *Ifng* reverse: 5′-ACAGCACAGGGAGCCTTTGT-3′. Reactions for each sample were performed in triplicate using an ABI StepOnePlus Detection System (Applied Biosystems, Foster City, CA) and a PCR protocol comprising an initial 10-min incubation at 95 °C followed by 40 cycles of 15 s at 95 °C and 1 min at 60–65 °C. The raw data were analyzed using StepOnePlus software (Applied Biosystems), and ∆∆Ct values for each gene in each sample were determined.

### Statistical analysis

Unpaired Student’s *t* test and one-way ANOVA were utilized as indicated and generated by GraphPad Prism 6 (version 6.0e).

## Results

### STAT4 regulates CD4 T cell production of GM-CSF during EAE

**Fig. 1:**
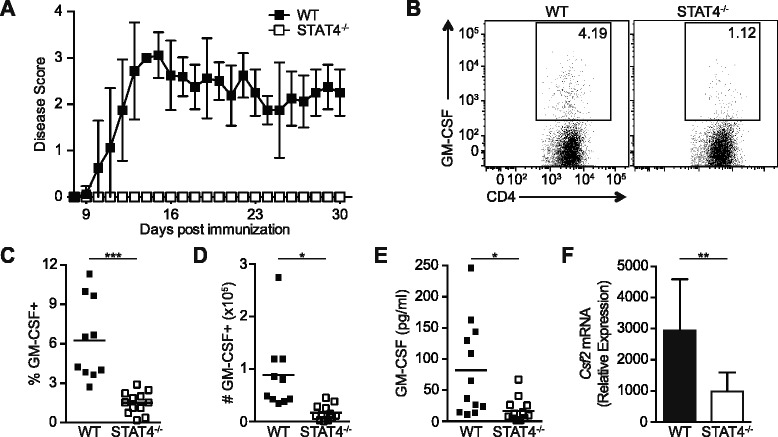
STAT4 is required for disease induction and GM-CSF production following EAE immunization. EAE was induced in WT and STAT4^−/−^ mice by MOG_35–55_ peptide immunization. **a** Mice were scored daily for disease severity. **b**–**f** Single-cell suspensions were prepared from the dLNs 10 days after EAE immunization and analyzed for GM-CSF production following MOG_35–55_ restimulation. **b** Representative plots gated on CD44^hi^CD4+ T cells. The cumulative (**c**) frequencies and (**d**) numbers of GM-CSF+ CD44^hi^CD4+ T cells are shown. **e** GM-CSF levels in culture supernatants were determined by ELISA. **f** GM-CSF mRNA expression was assayed in purified CD4 T cells. Data represent three independent experiments with (**a**) 2–5, (**b**–**e**) 3–6, or (**f**) 4–5 (pooled) mice in each group (mean ± SD). Student’s *t* test was performed: *p < 0.05; **p < 0.01; ***p < 0.001

Th1 and Th17 CD4 T cell subsets are important for inducing and maintaining EAE; however, the cardinal cytokines IFNγ and IL-17A are not required for pathogenesis [3–5, 15, 21]. Interestingly, the Th1-associated transcription factor STAT4 is necessary for EAE independent of the classical IL-12/IFNγ Th1 pathway, highlighting an unknown role of STAT4 during EAE [9–15]. In order to investigate this, we analyzed CD4 T cells from C57BL/6 (WT) and STAT4-deficient (STAT4^−/−^) mice immunized for EAE. In agreement with published reports [12, 14], we find that STAT4^−/−^ mice are resistant to EAE induction (Fig. 1a). Protection is not a result of increased frequency or number of Foxp3+ regulatory CD4 T cells (Additional file 1: Figure S1A). Furthermore, administration of an anti-IL-10R mAb during EAE did not restore disease susceptibility in STAT4^−/−^ mice, suggesting that STAT4 regulates EAE pathogenicity via pro-inflammatory mechanisms and not by the increase of anti-inflammatory IL-10 production (Additional file 1: Figure S1B).

Recent reports have indicated that GM-CSF is critical for EAE induction and is produced by both Th1 and Th17 subsets [22, 23]. Th1 differentiation is linked to STAT4, while in vitro Th17 development is independent of STAT4. Therefore, we postulated that STAT4 regulates Th1, but not Th17, GM-CSF production by effector CD4 T cells, which in turn determines encephalogenicity. To interrogate the regulation of GM-CSF by STAT4 during EAE, cells from the draining lymph nodes (dLN) were restimulated ex vivo with MOG_35−55_ peptide at the onset of disease (Fig. 1b). Both the frequency and number of GM-CSF+ CD4 T cells were significantly higher in the WT mice compared to the STAT4^−/−^ mice (Fig. 1b–d). In addition, GM-CSF secretion as well as mRNA levels were reduced in the STAT4^−/−^ cells compared to WT cells (Fig. 1e, f). These results demonstrate that STAT4 regulates GM-CSF production by MOG-specific CD4 T cells, signifying a potential pathogenic link between STAT4 and GM-CSF.

### Both Th1 and Th17 effector cells require STAT4 for GM-CSF production

**Fig. 2:**
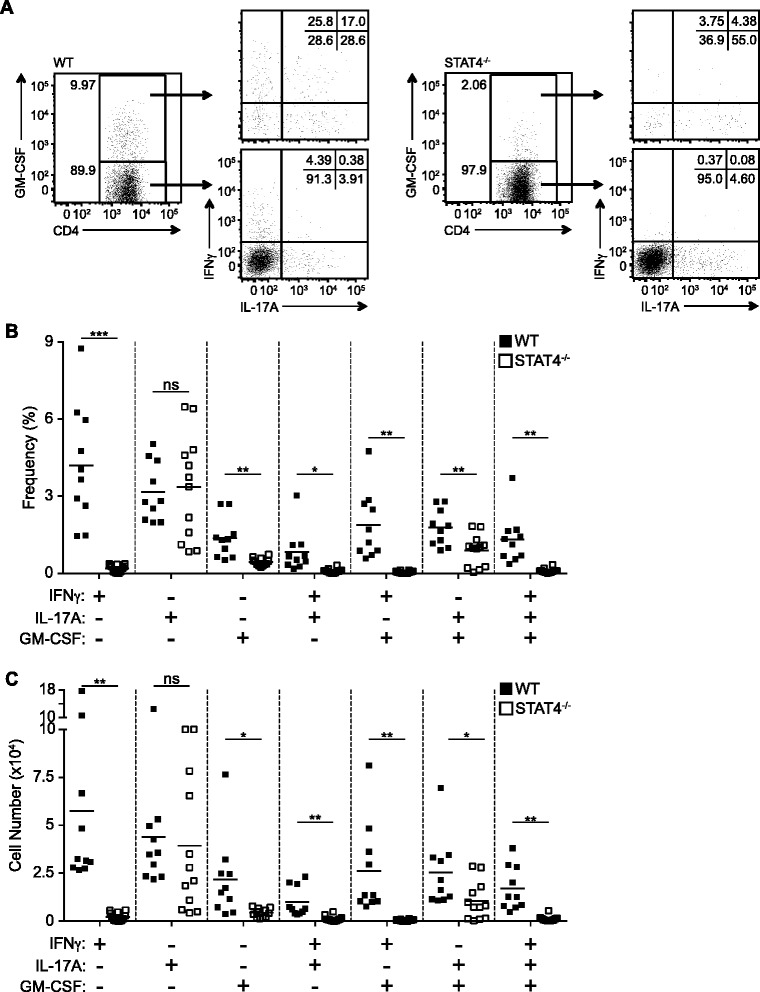
Th1 and Th17 CD4 T cell production of GM-CSF is dependent on STAT4 during EAE*.* MOG_35–55_ specific cytokine production was assessed in the dLN from WT and STAT4^−/−^ mice 10 days after EAE induction. **a** Representative plots are gated on CD44^hi^CD4+ T cells, and either GM-CSF+ (*top*) or GM-CSF− (*bottom*) CD4+ T cells. The (**b**) frequencies and (**c**) numbers of single, double, and triple cytokine-producing CD44^hi^CD4+ T cells are shown. Data represent three independent experiments with 3–5 mice in each group. Student’s *t* test was performed: ns = not significant; *p < 0.05; **p < 0.01; ***p < 0.001

**Fig. 3:**
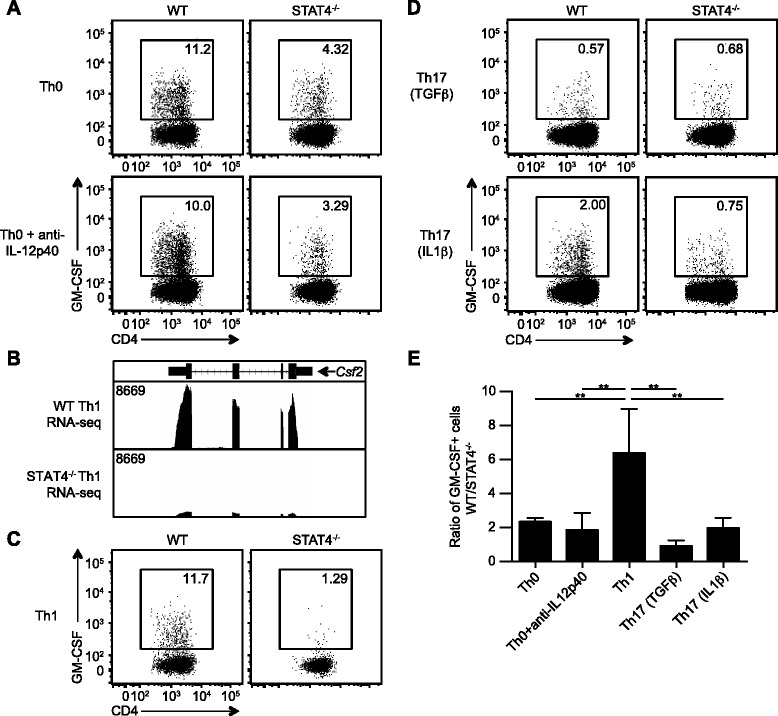
STAT4 regulates GM-CSF production in a cell-intrinsic, lineage-independent manner. Naïve CD4 T cells from WT and STAT4^−/−^ mice were activated under the indicated conditions for 6 days and then restimulated to assess GM-CSF production. **a** Representative GM-CSF staining gated on CD44^hi^CD4+ T cells for non-polarizing conditions are shown. **b** Publically available RNA-seq tracks comparing *Csf2* transcripts from WT [Geo: GSM994535] and STAT4^−/−^ [Geo: GSM994536] Th1 polarized cells, visualized using the IGV genome browser MM9 mouse gene database [17]. *Arrows* indicates gene direction. **c**–**d** Representative GM-CSF staining gated on CD44^hi^CD4+ T cells from **c** Th1 polarized and **d** Th17 polarized cells. **e** The ratio of GM-CSF+ CD44^hi^CD4+ T cells in the WT versus STAT4^−/−^ cultures was quantitated. Data represent three independent experiments (mean ± SD). One-way ANOVA with Tukey’s multiple comparisons test was performed: **p < 0.01

Our data indicate that STAT4 is necessary for robust GM-CSF production by activated CD4 T cells during EAE; however, both Th1 and Th17 subsets are capable of producing GM-CSF [22, 23]. Previous reports have demonstrated that Th17 cells develop independently of STAT4 [33], yet the role of STAT4 in regulating GM-CSF induction by these cells during EAE has not been investigated. To determine if STAT4 regulation of GM-CSF is restricted to a specific subset of effector CD4 T cells, we examined the MOG-specific cytokine response by CD4 T cells 10 days post EAE induction. In WT mice, we detected MOG-specific CD4 T cells capable of producing all combinations of IFNγ, IL-17A, and GM-CSF (single cytokine producers, double cytokine producers, and triple cytokine producers) (Fig. 2a). In mice lacking STAT4, we observed a significant reduction in the frequencies and numbers of IFNγ+ single and IFNγ+IL-17A+ double-producing CD4 T cells (Fig. 2b, c). Of note, there was a marked reduction in Th1-like IFNγ+GM-CSF+ double and IFNγ+IL-17A+GM-CSF+ triple-producing CD4 T cells. Moreover, consistent with previous reports [26, 27, 33], we did not note any differences in the IL-17A+ single-producing CD4 T cell populations between the WT and STAT4^−/−^ mice. However, cytokine analysis revealed a critical role for STAT4 in regulating GM-CSF production by Th17 cells as well as a unique subset of GM-CSF only producing cells; the frequencies and numbers of MOG-specific CD4 T cells that were GM-CSF+ single producers and IL-17A+GM-CSF+ double producers were reduced in the STAT4^−/−^ mice compared to WT mice (Fig. 3b, c). Taken together, these data signify that STAT4 does not solely function in Th1 cells during EAE, but acts broadly in a lineage-indiscriminant manner.

### STAT4 controls GM-CSF production in a cell-intrinsic, lineage-non-specific manner

STAT4 is important for GM-CSF production by effector CD4 T cells during EAE [14, 26]; however, these experiments do not define if STAT4 is functioning in a cell-intrinsic manner. To elucidate the CD4 T cell-intrinsic role of STAT4 in GM-CSF production, naïve CD4 T cells from WT and STAT4^−/−^ mice were activated in vitro for 6 days under non-polarizing, Th0 conditions. A marked population of GM-CSF+ cells was noted in the WT CD4 T cell cultures, and there was a consistent twofold reduction in the frequency of GM-CSF+ CD4 T cells if the cells lacked STAT4, indicating this molecule is operating intrinsically to CD4 T cells to regulate GM-CSF (Fig. 3a). Interestingly, our data suggests that under these conditions, STAT4 is not functioning downstream of IL-12 or IL-23, two cytokines associated with EAE and known to activate STAT4 [9–11, 34–37], as neutralization of IL-12/IL-23p40 had no impact on the in vitro differentiation of GM-CSF+ cells (Fig. 3a).

Th1 cells can be differentiated in vitro by the addition of IL-12, which subsequently signals via STAT4. To test the requirement of STAT4 for GM-CSF production by this subset of effector cells, naïve CD4 T cells from WT and STAT4^−/−^ mice were activated in vitro under Th1 polarizing conditions. Previously published and publically available data (GSE40463) indicated differences between Th1 polarized WT and STAT4^−/−^ CD4 T cells in *Csf2* RNA transcripts (Fig. 3b) [17]. Consistent with their data, we detected the highest amount of GM-CSF production by WT Th1 cells, and this was almost entirely STAT4 dependent, with a sixfold reduction in GM-CSF+ cells noted (Fig. 3c, e).

The generation of traditional Th17 cells in vitro with TGFβ1, IL-6, and IL-23 promotes effector cells that produce lower levels of GM-CSF and are less encephalogenic upon adoptive transfer compared to alternative Th17 cells differentiated in the presence of IL-1β, IL-6, and IL-23 [38, 39]. Hence, the Th17 polarizing conditions employed can impact the propensity of these cells to secrete GM-CSF. Traditional Th17 cells (TGFβ1) produced minimal GM-CSF regardless of the presence of STAT4 (Fig. 3d). In contrast, alternative Th17 cells (IL-1β) had increased frequencies of GM-CSF+ cells, and these cells were dependent on STAT4 signaling, as STAT4^−/−^ CD4 T cells consistently showed a twofold reduction in the frequency of GM-CSF+ cells (Fig. 3d, e). Together, these in vitro studies indicate that STAT4 functions in both a CD4 T cell-intrinsic and lineage-non-specific manner.

**Fig. 4:**
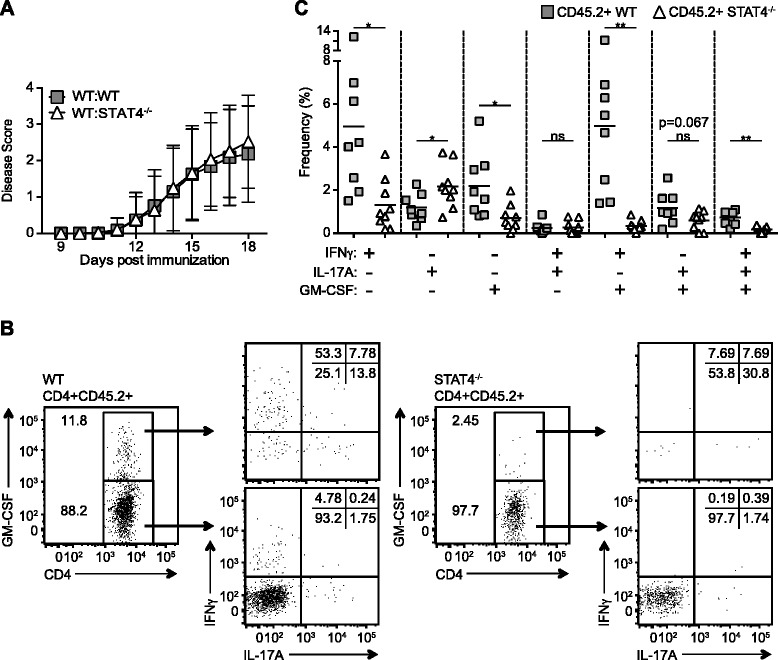
CD4 T cell-intrinsic expression of STAT4 is critical for production of GM-CSF during EAE. EAE was induced in WT:WT and WT:STAT4^−/−^ mixed bone marrow chimeric mice. **a** Mice were scored daily for disease severity. **b**–**c** Spinal cord infiltrating lymphocytes were restimulated with MOG_35–55_ peptide and analyzed for IFNγ, IL-17A, and GM-CSF production. **b** Representative plots are gated on CD4+CD45.2+ T cells as well as GM-CSF+ (*top*) or GM-CSF− (*bottom*) cells. **c** The cumulative frequencies of IFNγ, IL-17A, and GM-CSF single, double, and triple cytokine-producing CD4+CD45.2+ T cells are shown. Data represent 2–7 independent experiments with (**a**) 3–5 or (**b**–**c**) 4–5 mice in each group (mean ± SD). Student’s *t* test was performed: ns = not significant; *p < 0.05; **p < 0.01

To verify that STAT4 operates in a CD4 T cell-intrinsic mode to regulate GM-CSF production during EAE, we generated mixed bone marrow chimeric mice consisting of 50 % CD45.1 WT bone marrow and 50 % CD45.2 STAT4^−/−^ bone marrow. We also made mixed bone marrow chimeric mice with 50 % CD45.1 WT bone marrow and 50 % CD45.2 WT bone marrow for control purposes. Both cohorts of mice demonstrated similar disease onset and severity following EAE induction (Fig. 4a). At the peak of EAE disease, day 17, cells from the spinal cords of diseased mice were assayed for GM-CSF, as well as IFNγ and IL-17A production, by CD45.2+ CD4 T cells after ex vivo MOG_35−55_ peptide restimulation. Similar to the intact mice, we observed an appreciable population of WT GM-CSF+ CD4 T cells; however, the frequency of STAT4^−/−^ CD4 T cells producing GM-CSF was markedly reduced (Fig. 4b, c). There was also a significant decrease in the percentage of STAT4^−/−^ CD4 T cells that were IFNγ+GM-CSF+ double producers and IFNγ+IL-17A+GM-CSF+ triple producers. We noted a decline in the frequency of STAT4^−/−^ CD4 T cells co-producing IL-17A and GM-CSF; however, this did not reach statistical significance. Interestingly, we did detect an increase in the proportion of STAT4^−/−^ CD4 T cells producing IL-17A only, which further emphasizes a previously undescribed role for STAT4 in regulating Th17 function, including the modulation of GM-CSF levels in these cells.

### STAT4 directly interacts with the Csf2 locus to activate transcription

**Fig. 5:**
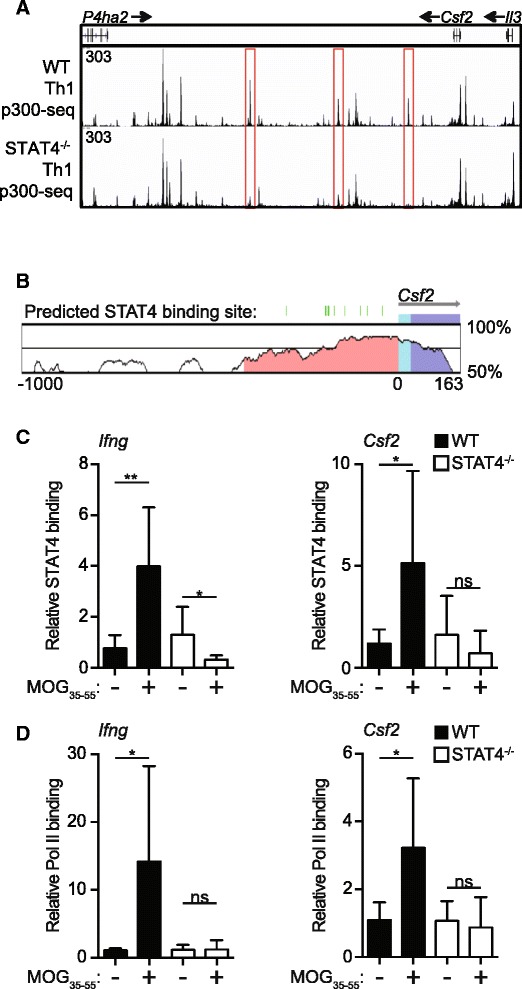
STAT4 interacts with the *Csf2* promoter and correlates with activation. **a** Publically available p300 ChIP-seq tracks from WT [Geo: GSM994508] and STAT4^−/−^ [Geo: GSM994509] Th1 polarized cells were visualized for the *Csf2* gene loci using IGV genome browser mouse MM9 database [17]. *Arrows* denote gene direction. **b** Conservation of the *Csf2* promoter region between human and mouse. Predicted STAT4 binding sites are indicated by the *green lines*. Schematic and data were generated for this region by the rVista2.0 program (http://pipeline.lbl.gov/). **c**–**d** CD4 T cells from the spleens and dLNs of WT and STAT4^−/−^ mice that had been previously immunized for EAE 10 days earlier were stimulated with MOG_35–55_ peptide and analyzed by ChIP. **c** STAT4 and (**d**) Pol II interactions with the *Ifng* and *Csf2* promoter regions were assessed. Data are normalized to input control and quantitated relative to media stimulation. Data represent three independent experiments with 4–5 (pooled) mice in each group (mean ± SD). Student’s *t* test was performed: ns = not significant; *p < 0.05

Both in vitro and in vivo, we find that STAT4 signaling is critical for optimal lineage-non-specific GM-CSF expression; however, these data do not reveal if STAT4 is acting directly to modify the *Csf2* locus and/or activate gene transcription. The *Csf2* upstream promoter region is highly conserved between mouse and human, and several predicted STAT4 binding sites are present in this region [40]. Further, publically available data (GSE40463) assessing the binding of p300 in WT and STAT4^−/−^ Th1 polarized cells indicate that STAT4 regulates several enhancer regions around the *Csf2* locus, suggesting that STAT4 has direct effects on the production of GM-CSF (Fig. 5a) [17]. To determine if STAT4 is directly regulating GM-CSF, we performed ChIP analysis of the 1000 bp region upstream of *Csf2* promoter which has predicted STAT4 binding sites (Fig. 5b). WT and STAT4^−/−^ mice were immunized for EAE, and on day 10, CD4 T cells from the spleen and dLN were pooled and activated with the MOG_35−55_ peptide for 5 h. This short period of antigen reactivation is sufficient to induce transcription of cytokine genes prior to the onset of cell proliferation. Using ChIP-PCR, we detected significant induction of STAT4 binding to the *Csf2* promoter in the WT CD4 T cells stimulated with the MOG peptide compared to unstimulated CD4 T cells (Fig. 5c). As anticipated, STAT4 binding to the *Ifng* promoter was also increased after MOG stimulation in WT CD4 T cells and no STAT4 binding to the *Csf2* or *Ifng* promoters was noted in STAT4^−/−^ CD4 T cells. Importantly, Pol II binding to the *Csf2* promoter was increased in WT CD4 T cells following MOG stimulation, but induction of Pol II binding was not observed in STAT4^−/−^ CD4 T cells, indicating that transcription of the *Csf2* gene is reduced in the absence of STAT4 (Fig. 5d). Taken together, these data indicate that STAT4 functions to promote GM-CSF production via a direct interaction with the *Csf2* locus.

## Discussion

STAT4 is a transcription factor necessary for the differentiation of the Th1 lineage of effector CD4 T cells [7, 8]. However, while STAT4 is critical for EAE, neither the upstream STAT4 activating cytokine IL-12 nor the downstream Th1 effector cytokine IFNγ are needed for disease induction [9–15]. This implicates a role for STAT4 outside of the traditional Th1-associated IL-12 signaling pathway. We find that, when activated in vitro under non-polarizing conditions, the absence of STAT4 results in the decreased ability of CD4 T cells to produce GM-CSF, whereas blocking IL-12 has minimal effect on GM-CSF production by CD4 T cells. This is consistent with published data, as well as the data presented herein, showing CD4 T cell production of GM-CSF during EAE is independent of IL-12 signaling but dependent on STAT4 expression. Our findings do not negate the ability of IL-12 to induce GM-CSF in a STAT4-dependent manner, but do indicate that additional molecules signal via STAT4 to promote GM-CSF expression. Taken together, these data demonstrate STAT4 can function to regulate GM-CSF separately from the classic Th1 pathway, particularly during EAE. In keeping with these data, our study shows a marked decrease in Th1-like IFNγ+GM-CSF+, Th17-like IL-17A+GM-CSF+, and GM-CSF+ single-producing CD4 T cells, indicating that STAT4 may be a central regulator of GM-CSF expression in a lineage-indiscriminant manner.

CD4 T cells co-producing IFNγ and IL-17A are present during EAE and are postulated to be highly pathogenic [18, 41]. Our interrogation of GM-CSF expression by effector CD4 T cells revealed that the majority of the IFNγ+IL-17A+ double-producing cells in WT mice during EAE concurrently express GM-CSF, and this may explain the pathogenic propensity of this cell population. The IFNγ+IL-17A+ double-producing cells have been shown to arise from Th17 CD4 T cells [41], inferring that the triple cytokine-producing cells are also derived from plastic Th17 cells. The role of STAT4 in Th17 plasticity is debatable; the emergence of IFNγ+IL-17A+ cells from Th17 cells after repeated IL-23 stimulation in vitro has been shown to be both dependent on, as well as independent of, STAT4 [18, 19]. Interestingly, we show that STAT4^−/−^ CD4 T cells lack the IFNγ+IL-17A+GM-CSF+ triple cytokine-producing cell population; thus, the requirement of STAT4 to induce CNS inflammation may be linked to Th17 plasticity and the development of this particular effector cell subset.

The production of IL-17A by Th17 cells has been shown to be independent of STAT4 and, in fact, we demonstrate that during EAE, the frequency of IL-17A+ CD4 T cells is actually increased in the absence of STAT4. One potential explanation for this observation is the role of STAT4 in modulating expression of the Th1 master transcription factor, Tbet. In the absence of STAT4, Tbet is induced, but not to the same levels as in WT CD4 T cells (McWilliams, data not shown). Tbet can repress IL-17A production [42, 43]; hence, the effect of STAT4 may be a consequence of lower Tbet levels. Similarly, the Th1 cytokine IFNγ is able to suppress IL-17A secretion by CD4 T cells [31, 33] and STAT4 is necessary for optimal expression of IFNγ [44]; therefore, the increase in IL-17A+ CD4 T cells may be the result of diminished autocrine IFNγ signaling. Another possible reason for the augmented IL-17A frequencies in the absence of STAT4 is the inability of these cells to undergo Th17 plasticity [19]. Th17 cells that are unable to convert into IL-17A+IFNγ+, and potentially then IFNγ+ CD4 T cells, may yield higher percentages of these cells during EAE.

Both GM-CSF single and triple cytokine-producing CD4 T cells were recently described in MS patients [40], but the ontogeny of these effector cell populations and how these cells contribute to MS and EAE pathology remain unclear. In this study, we show that there is a marked reduction not only in IFNγ+IL-17A+GM-CSF+ triple-producing CD4 T cells in the absence of STAT4 during EAE, but in all GM-CSF producing effector CD4 T cells. This includes GM-CSF single-producing cells as well as IL-17A+GM-CSF+ double-producing cells, which would presumably be Th17 cells. Therefore, STAT4 regulates GM-CSF production in various subsets of effector CD4 T cells, and the decreased production of GM-CSF by STAT4^−/−^ CD4 T cells is not solely the result of impaired Th17 plasticity. It is unclear if the same molecule mediates the development of these different GM-CSF-producing CD4 T cell populations. One cytokine shown to regulate GM-CSF production in mice is IL-23 [22, 23, 45, 46], and it is interesting to speculate that the impaired GM-CSF production by STAT4^−/−^ CD4 T cells is linked to defective IL-23 signaling. Importantly, data from our lab demonstrates that IL-23-induced STAT3 activation occurs independent of STAT4 expression during EAE (McWilliams, manuscript submitted), indicating that the predominant IL-23 signaling pathway is intact in the absence of STAT4. Together, these data suggest that the function of STAT4 in the formation of GM-CSF single, double, and triple cytokine-producing CD4 T cells may be separate from the role of IL-23 in the expression of this cytokine during EAE and that a potentially novel STAT4 ligand drives CNS inflammation.

We show that during EAE, STAT4 directly interacts with the *Csf2* gene locus to regulate optimal GM-CSF expression. It is well documented that STAT4 controls the accessibility of multiple Th1-associated genes; however, these studies did not highlight GM-CSF as a STAT4 regulated target [7, 16, 17]. The discrepancies in these data may reflect differences in the CD4 T cell populations examined or the manner in which the cells were activated; previous reports have studied IL-12-induced STAT4 gene regulation in differentiating Th1 cells, whereas we examined the role of STAT4 in GM-CSF production by bulk effector CD4 T cells during EAE, which is independent of IL-12. These data imply there exists a set of unidentified STAT4-dependent genes present in vivo during autoimmunity and possibly other inflammatory conditions. Deciphering what these genes are and which molecules operate via STAT4 to promote gene expression during disease will be important for dissecting the underlying causes of autoimmune inflammation.

Our study identifies STAT4 as a potent regulator of GM-CSF production by effector CD4 T cells of various lineages. Nevertheless, while GM-CSF levels are significantly decreased in STAT4^−/−^ CD4 T cells, this cytokine is still detectable suggesting that other transcription factors must mediate expression. Recent publications have identified both IL-2 and IL-7, signaling via STAT5, as additional regulators of T cell-derived GM-CSF [40, 47]. This raises an interesting question as to the respective contributions of STAT4 and STAT5 to GM-CSF expression and if these molecules act cooperatively to promote optimal GM-CSF production. One proposed function of the STAT transcription factors is to modulate the accessibility of lineage-specific genes [7, 16, 17]. Additionally, STAT4 alters the enhancer landscape around the *Csf2* locus [17]. Therefore, a plausible explanation as to how STAT4 and STAT5 may both regulate GM-CSF is that STAT4 is controlling the accessibility or optimal transcription conditions of the *Csf2* locus for STAT5 involvement. Conversely, STAT5 may be necessary to open the *Csf2* locus in order for STAT4 to then interact, as we have shown that STAT4 is able to directly bind to the *Csf2* promoter after MOG stimulation. Future studies will be necessary to address this as well as to determine if STAT4 expression is critical for the ability of other transcription factors to bind and promote GM-CSF production in effector CD4 T cells.

## Conclusions

This study identifies a previously unrecognized role for STAT4 to directly bind to the *Csf2* promoter and control GM-CSF expression in CD4 T cells during neuronal inflammation. Further, our data challenges the previously held Th1-centric model of STAT4 activity by demonstrating that during EAE, STAT4 functions within Th17 cells to modulate GM-CSF levels. Together, our data suggests that STAT4 may be an attractive therapeutic target in MS; however, additional research needs to be performed to assess this possibility.

## Additional file

Additional file 1: Figure S1. STAT4 deletion does not increase the presence or function of Tregs. (A) WT and STAT4^−/−^ mice were immunized for EAE. The frequencies and numbers of Foxp3+CD25+CD4+ T cells were determined in the dLN 10 days post immunization. (B) Disease severity was monitored in WT and STAT4^−/−^ mice following EAE immunization and subsequent PBS or anti-IL-10R mAb treatment. Data represent three independent experiments with (A) 3–6 and (B) 2–5 mice per group. Student’s *t* test was performed: ns = not significant; **p* < 0.05.

## References

[1] Sospedra M, Martin R (2005). Immunology of multiple sclerosis. Annu Rev Immunol.

[2] Arnason BG (1999). Immunologic therapy of multiple sclerosis. Annu Rev Med.

[3] Pierson E, Simmons SB, Castelli L, Goverman JM (2012). Mechanisms regulating regional localization of inflammation during CNS autoimmunity. Immunol Rev.

[4] Steinman L (2007). A brief history of T(H)17, the first major revision in the T(H)1/T(H)2 hypothesis of T cell-mediated tissue damage. Nat Med.

[5] Goverman J (2009). Autoimmune T, cell responses in the central nervous system. Nat Rev Immunol.

[6] Beecham AH, Patsopoulos NA, Xifara DK, Davis MF, Kemppinen A, Cotsapas C (2013). Analysis of immune-related loci identifies 48 new susceptibility variants for multiple sclerosis. Nat Genet.

[7] Good SR, Thieu VT, Mathur AN, Yu Q, Stritesky GL, Yeh N (2009). Temporal induction pattern of STAT4 target genes defines potential for Th1 lineage-specific programming. J Immunol.

[8] Trinchieri G (2003). Interleukin-12 and the regulation of innate resistance and adaptive immunity. Nat Rev Immunol.

[9] Zhang GX, Gran B, Yu S, Li J, Siglienti I, Chen X (2003). Induction of experimental autoimmune encephalomyelitis in IL-12 receptor-beta 2-deficient mice: IL-12 responsiveness is not required in the pathogenesis of inflammatory demyelination in the central nervous system. J Immunol.

[10] Cua DJ, Sherlock J, Chen Y, Murphy CA, Joyce B, Seymour B (2003). Interleukin-23 rather than interleukin-12 is the critical cytokine for autoimmune inflammation of the brain. Nature.

[11] Gran B, Zhang GX, Yu S, Li J, Chen XH, Ventura ES (2002). IL-12p35-deficient mice are susceptible to experimental autoimmune encephalomyelitis: evidence for redundancy in the IL-12 system in the induction of central nervous system autoimmune demyelination. J Immunol.

[12] Chitnis T, Najafian N, Benou C, Salama AD, Grusby MJ, Sayegh MH (2001). Effect of targeted disruption of STAT4 and STAT6 on the induction of experimental autoimmune encephalomyelitis. J Clin Invest.

[13] Bright JJ, Du C, Sriram S (1999). Tyrphostin B42 inhibits IL-12-induced tyrosine phosphorylation and activation of Janus kinase-2 and prevents experimental allergic encephalomyelitis. J Immunol.

[14] Mo C, Chearwae W, O'Malley JT, Adams SM, Kanakasabai S, Walline CC (2008). Stat4 isoforms differentially regulate inflammation and demyelination in experimental allergic encephalomyelitis. J Immunol.

[15] Lovett-Racke AE, Yang Y, Racke MK (2011). Th1 versus Th17: are T cell cytokines relevant in multiple sclerosis?. Biochim Biophys Acta.

[16] Wei L, Vahedi G, Sun HW, Watford WT, Takatori H, Ramos HL (2010). Discrete roles of STAT4 and STAT6 transcription factors in tuning epigenetic modifications and transcription during T helper cell differentiation. Immunity.

[17] Vahedi G, Takahashi H, Nakayamada S, Sun HW, Sartorelli V, Kanno Y (2012). STATs shape the active enhancer landscape of T cell populations. Cell.

[18] Duhen R, Glatigny S, Arbelaez CA, Blair TC, Oukka M, Bettelli E (2013). Cutting edge: the pathogenicity of IFN-gamma-producing Th17 cells is independent of T-bet. J Immunol.

[19] Lee YK, Turner H, Maynard CL, Oliver JR, Chen D, Elson CO (2009). Late developmental plasticity in the T helper 17 lineage. Immunity.

[20] Mukasa R, Balasubramani A, Lee YK, Whitley SK, Weaver BT, Shibata Y (2010). Epigenetic instability of cytokine and transcription factor gene loci underlies plasticity of the T helper 17 cell lineage. Immunity.

[21] Kroenke MA, Chensue SW, Segal BM (2010). EAE mediated by a non-IFN-gamma/non-IL-17 pathway. Eur J Immunol.

[22] Codarri L, Gyulveszi G, Tosevski V, Hesske L, Fontana A, Magnenat L (2011). RORgammat drives production of the cytokine GM-CSF in helper T cells, which is essential for the effector phase of autoimmune neuroinflammation. Nat Immunol.

[23] El-Behi M, Ciric B, Dai H, Yan Y, Cullimore M, Safavi F (2011). The encephalitogenicity of T(H)17 cells is dependent on IL-1- and IL-23-induced production of the cytokine GM-CSF. Nat Immunol.

[24] Hamilton JA (2008). Colony-stimulating factors in inflammation and autoimmunity. Nat Rev Immunol.

[25] Ponomarev ED, Shriver LP, Maresz K, Pedras-Vasconcelos J, Verthelyi D, Dittel BN (2007). GM-CSF production by autoreactive T cells is required for the activation of microglial cells and the onset of experimental autoimmune encephalomyelitis. J Immunol.

[26] Pham D, Yu Q, Walline CC, Muthukrishnan R, Blum JS, Kaplan MH (2013). Opposing roles of STAT4 and Dnmt3a in Th1 gene regulation. J Immunol.

[27] O'Malley JT, Eri RD, Stritesky GL, Mathur AN, Chang HC, Hogenesch H (2008). STAT4 isoforms differentially regulate Th1 cytokine production and the severity of inflammatory bowel disease. J Immunol.

[28] Kaplan MH, Sun YL, Hoey T, Grusby MJ (1996). Impaired IL-12 responses and enhanced development of Th2 cells in Stat4-deficient mice. Nature.

[29] Harrington LE, Janowski KM, Oliver JR, Zajac AJ, Weaver CT (2008). Memory CD4 T cells emerge from effector T-cell progenitors. Nature.

[30] Yi JS, Du M, Zajac AJ (2009). A vital role for interleukin-21 in the control of a chronic viral infection. Science.

[31] Yeh WI, McWilliams IL, Harrington LE (2011). Autoreactive Tbet-positive CD4 T cells develop independent of classic Th1 cytokine signaling during experimental autoimmune encephalomyelitis. J Immunol.

[32] Nozell S, Laver T, Moseley D, Nowoslawski L, De Vos M, Atkinson GP (2008). The ING4 tumor suppressor attenuates NF-kappaB activity at the promoters of target genes. Mol Cell Biol.

[33] Harrington LE, Hatton RD, Mangan PR, Turner H, Murphy TL, Murphy KM (2005). Interleukin 17-producing CD4+ effector T cells develop via a lineage distinct from the T helper type 1 and 2 lineages. Nat Immunol.

[34] Kroenke MA, Carlson TJ, Andjelkovic AV, Segal BM (2008). IL-12- and IL-23-modulated T cells induce distinct types of EAE based on histology, CNS chemokine profile, and response to cytokine inhibition. J Exp Med.

[35] McGeachy MJ, Chen Y, Tato CM, Laurence A, Joyce-Shaikh B, Blumenschein WM (2009). The interleukin 23 receptor is essential for the terminal differentiation of interleukin 17-producing effector T helper cells in vivo. Nat Immunol.

[36] de Paus RA, van de Wetering D, van Dissel JT, van de Vosse E (2008). IL-23 and IL-12 responses in activated human T cells retrovirally transduced with IL-23 receptor variants. Mol Immunol.

[37] Oppmann B, Lesley R, Blom B, Timans JC, Xu Y, Hunte B (2000). Novel p19 protein engages IL-12p40 to form a cytokine, IL-23, with biological activities similar as well as distinct from IL-12. Immunity.

[38] Ghoreschi K, Laurence A, Yang XP, Tato CM, McGeachy MJ, Konkel JE (2010). Generation of pathogenic T(H)17 cells in the absence of TGF-beta signalling. Nature.

[39] Lee Y, Awasthi A, Yosef N, Quintana FJ, Xiao S, Peters A (2012). Induction and molecular signature of pathogenic TH17 cells. Nat Immunol.

[40] Noster R, Riedel R, Mashreghi MF, Radbruch H, Harms L, Haftmann C (2014). IL-17 and GM-CSF expression are antagonistically regulated by human T helper cells. Sci Transl Med.

[41] Hirota K, Duarte JH, Veldhoen M, Hornsby E, Li Y, Cua DJ (2011). Fate mapping of IL-17-producing T cells in inflammatory responses. Nat Immunol.

[42] Yeh WI, McWilliams IL, Harrington LE (2014). IFNgamma inhibits Th17 differentiation and function via Tbet-dependent and Tbet-independent mechanisms. J Neuroimmunol.

[43] Lazarevic V, Chen X, Shim JH, Hwang ES, Jang E, Bolm AN (2011). T-bet represses T(H)17 differentiation by preventing Runx1-mediated activation of the gene encoding RORgammat. Nat Immunol.

[44] Kaplan MH, Wurster AL, Grusby MJ (1998). A signal transducer and activator of transcription (Stat)4-independent pathway for the development of T helper type 1 cells. J Exp Med.

[45] McGeachy MJ (2011). GM-CSF: the secret weapon in the T(H)17 arsenal. Nat Immunol.

[46] Becher B, Segal BM (2011). T(H)17 cytokines in autoimmune neuro-inflammation. Curr Opin Immunol.

[47] Sheng W, Yang F, Zhou Y, Yang H, Low PY, Kemeny DM (2014). STAT5 programs a distinct subset of GM-CSF-producing T helper cells that is essential for autoimmune neuroinflammation. Cell Res.

