# Supplementary material for: STAT4 controls GM-CSF production by both Th1 and Th17 cells during EAE
Source: J Neuroinflammation. 2015 Jun 30;12:128. doi: 10.1186/s12974-015-0351-3 (PMC4491892; doi:10.1186/s12974-015-0351-3)
Supplement: Additional file 1: Figure S1. — STAT4 deletion does not increase the presence or function of Tregs. (A) WT and STAT4−/− mice were immunized for EAE. The frequencies and numbers of Foxp3+CD25+CD4+ T cells were determined in the dLN 10 days post immunization. (B) Disease severity was monitored in WT and STAT4−/− mice following EAE immunization and subsequent PBS or anti-IL-10R mAb treatment. Data represent three independent experiments with (A) 3–6 and (B) 2–5 mice per group. Student’s t test was performed: ns = not significant; *p < 0.05. [file 12974_2015_351_MOESM1_ESM.pdf]

SUPPORTING INFORMATION

SI MATERIALS AND METHODS

FoxP3 *ex-vivo* analysis.

The following intracellular antibody was used in accordance with manufacturers protocols: anti-FoxP3 FITC (eBioscience clone: FJK-16s) and anti-CD25 PerCP-Cy5.5 (eBioscience, clone PC61.5).

Anti-IL-10R mAb blockade during EAE.

Anti-IL-10R blocking mAb treatment (clone 1B1.3a; UAB hybridoma facility) was administered by i.p. injection at 250μg per mouse on days 0, 7, 14, and 21 post-immunization.

SI FIGURE LEGENDS

Figure S1: STAT4 deletion does not increase the presence or function of Tregs. **(A)** WT and $STAT4^{-/-}$ mice were immunized for EAE. The frequencies and numbers of Foxp3+CD25+CD4+ T cells were determined in the dLN 10 days post immunization. **(B)** Disease severity was monitored in WT and $STAT4^{-/-}$ mice following EAE immunization and subsequent PBS or anti-IL-10R mAb treatment. Data represent 3 independent experiments with (A) 3-6 and (B) 2-5 mice per group. Student's t-test was performed: ns=not significant; *=p<0.05.

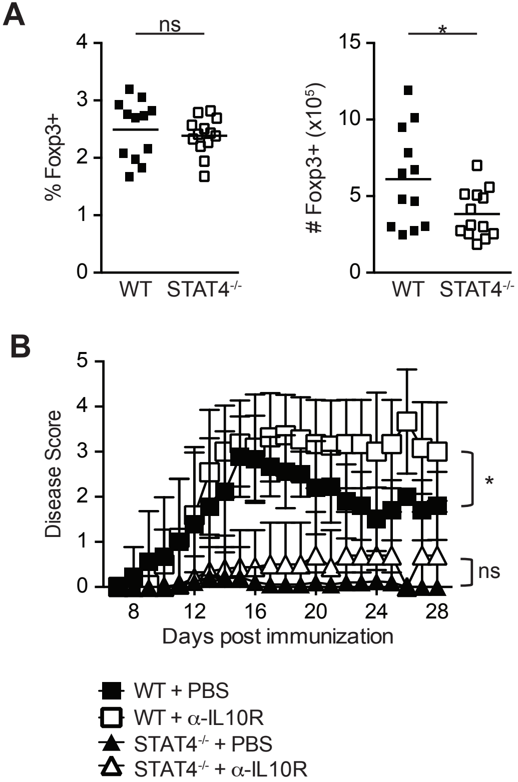
A
ns
*
% Foxp3+
# Foxp3+ (x10^5)
WT
STAT4-/-
B
Disease Score
Days post immunization
*
ns
WT + PBS
WT + α-IL10R
STAT4-/- + PBS
STAT4-/- + α-IL10R

SFigure 1
